# Direct Determination of a Small-Molecule Drug, Valproic Acid, by an Electrically-Detected Microcantilever Biosensor for Personalized Diagnostics

**DOI:** 10.3390/bios5010037

**Published:** 2015-01-27

**Authors:** Long-Sun Huang, Christian Gunawan, Yi-Kuang Yen, Kai-Fung Chang

**Affiliations:** 1 Institute of Applied Mechanics, National Taiwan University, Taipei 10617, Taiwan; E-Mails: cgwu@ntumems.net (C.G.); yi-kuang.yen@weizmann.ac.il (Y.-K.Y.); kfjhang@ntumems.net (K.-F.C.); 2 Department of Chemical Physics, Weizmann Institute of Science, 234 Herzl Street, Rehovot 7610001, Israel

**Keywords:** valproic acid, microcantilever, serum, therapeutic drug monitoring

## Abstract

Direct, small-molecule determination of the antiepileptic drug, valproic acid, was investigated by a label-free, nanomechanical biosensor. Valproic acid has long been used as an antiepileptic medication, which is administered through therapeutic drug monitoring and has a narrow therapeutic dosage range of 50–100 μg·mL^−^^1^ in blood or serum. Unlike labeled and clinically-used measurement techniques, the label-free, electrical detection microcantilever biosensor can be miniaturized and simplified for use in portable or hand-held point-of-care platforms or personal diagnostic tools. A micromachined microcantilever sensor was packaged into the micro-channel of a fluidic system. The measurement of the antiepileptic drug, valproic acid, in phosphate-buffered saline and serum used a single free-standing, piezoresistive microcantilever biosensor in a thermally-controlled system. The measured surface stresses showed a profile over a concentration range of 50–500 μg·mL^−^^1^, which covered the clinically therapeutic range of 50–100 μg·mL^−^^1^. The estimated limit of detection (LOD) was calculated to be 45 μg·mL^−1^, and the binding affinity between the drug and the antibody was measured at around 90 ± 21 μg·mL^−^^1^. Lastly, the results of the proposed device showed a similar profile in valproic acid drug detection with those of the clinically-used fluorescence polarization immunoassay.

## 1. Introduction

Microcantilever biosensors have shown great promise for application in chemical and biological analyses [1]. The microcantilever biosensing technique demonstrated label-free, direct determination in DNA hybridization [2,3], antibody-antigen binding [4,5], enzyme reactions [6], drug screening [7] and drug detection [8]. The specific bindings of molecular interactions induce a biomolecular conformation change, resulting in a nanomechanical response or the deflection of a microcantilever.

Label-free detection eliminates the need for tags, dyes or specialized reagents and is thus of considerable interest for simplifying assay design and minimizing difficulties in the use of labels. In addition to not requiring fluorescence labeling, this biosensing technique provides high sensitivity and miniaturization for potential application in portable or disposable point-of-care platforms and personalized diagnostics.

Several label-free techniques have been developed for biosensor detection. In label-free quartz crystal microbalance (QCM) immunosensors based on frequency change, the technique mainly focuses on measurements in the dry state in air. For dip-and-dry procedures, the measurement involves processes that are relatively cumbersome, time consuming and prone to error, due to hydration and humidity [9,10]. Moreover, label-free optical surface plasmon resonance (SPR) biosensors that use refractive index changes to detect mass changes at sensor surface interfaces pose direct challenges for the detection of small molecules of less than 2 kDa, due to weak signals and poor sensitivity [11]. A competition-based immunoassay, which mixes unlabeled and high-mass nanoparticle-bound small molecules for interaction with antibodies, is introduced to detect targets with small molecular weights [11]. As for nanowire field effect transistor biosensors, the label-free sensing mechanism is based on the gating effect of the surface charge. Few studies have been reported, but a successful example of the binding/inhibition of negatively-charged ATP onto the tyrosine kinase Ab1 was demonstrated using the competitive small-molecule antagonist [12]. The use of microcantilever biosensors, especially for small-molecule detection, exhibits advantages over other label-free, affinity-based biosensors.

Small-molecule valproic acid is widely used to treat certain types of seizures or epilepsy [13]. Most antiepileptic drugs have narrow ranges of drug concentrations in serum or blood. Excessive drug concentrations in the blood may result in patient toxicity, while low drug concentrations can lead to treatment failure. The epileptic drug, valproic acid, has a narrow reference therapeutic concentration range of 50–100 μg·mL^−^^1^ in blood, requiring the administration of therapeutic drug monitoring (TDM).

Although TDM drugs list their recommended dosages, individual therapeutic concentration ranges must be personalized for each patient and, thus, may vary widely [14]. Valproic acid measurement is commonly and clinically performed through competition-based high-throughput fluorescence polarization immunoassays (FPIA). This technique requires the use of skilled technicians, milliliter-scale samples and reagent volumes and long turnaround times [15].

In this study, the electrical transducer of a microcantilever deflection is employed for miniaturization, while the optical-level measurement technique requires space and alignment for additional components, such as a laser, optics and detectors [1]. In an electrical transduction scheme, the dual-beam, free-standing microcantilevers in the Wheatstone bridge circuit have been extensively used for the detection of chemical interactions and biomolecular recognition in liquid environments [5]. In contrast to the reference cantilever, the sensing and recognition element-coated microcantilever exhibited a certain degree of sensitivity to pH, ionic or chemical solutions [16,17]. The single free-standing cantilever configuration is adopted to prevent unexpected or irreproducible responses [18].

This work presents a simple, point-of-care detection method for the antiepileptic drug, valproic acid, using a single free-standing, thermally-controlled piezoresistive microcantilever. Various concentrations of valproic acid are measured both in phosphate-buffered saline (PBS) and in serum of a complex liquid environment. Results are compared for the proposed microcantilever biosensor and clinically-used FPIA instruments.

## 2. Materials and Methods

### 2.1. Fabrication

**Figure 1:**
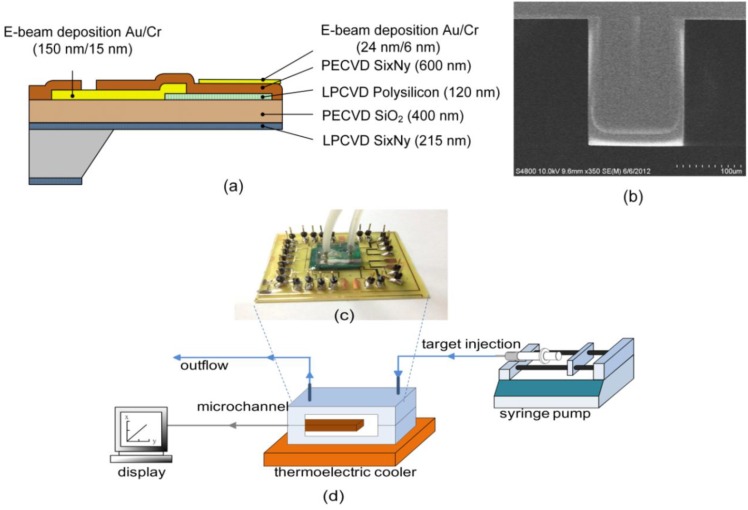
(**a**) Fabrication process of a piezoresistive microcantilever sensor; (**b**) an SEM image of a complete piezoresistive microcantilever biosensor; (**c**) a photograph of a packaged device; (**d**) an experimental system setup. PECVD, plasma-enhanced chemical vapor deposition; LPCVD, low-pressure chemical vapor deposition.

Figure 1a shows the microcantilever cross-sectional view and its associated processes [8]. The piezoresistive microcantilever is composed of several layers, including a structural insulation layer of nitride (Si_x_N_y_) and oxide, a piezoresistive polysilicon layer, an insulation layer of nitride and a gold layer for the surface reaction. A 500 µm-thick p-type <100> silicon wafer was used as a starting substrate. First, a 215-nm low-pressure chemical vapor deposition (LPCVD) low-stress nitride layer was deposited on both the top and bottom sides of a Si wafer at 780 °C. The top layer was used as a protective piezoresistive polysilicon layer in later KOH backside wet etching, and the bottom layer acted as a blocking mask for KOH wet etching. Then, a 400-nm SiO_2_ film was deposited on the Si_3_N_4_ top layer using a plasma-enhanced chemical vapor deposition (PECVD) technique to form an overall residual stress balance after release, allowing a released microcantilever to lie as flat as possible for minimum curvature, thus significantly reducing flow-induced disturbance. A 120-nm polysilicon layer was deposited by LPCVD, followed by a boron-doped ion implantation with energy of 20 keV, a doping concentration of 5.437 × 10^19^ cm^−3^ and at an annealing temperature of 1080 °C. Reactive-ion etching (RIE) was used to define the patterned polysilicon layer for a piezoresistive effect. Subsequently, 15-nm chromium (Cr) and 150-nm gold (Au) were evaporated on the polysilicon layer to be electrically connected for wire bonding. Meanwhile, a Cr layer served as an interfacial layer to re-form an adhesive force for the gold layer. A 600-nm plasma-enhanced chemical vapor deposition (PECVD) nitride film was used as a passivation layer to protect the structures etched using backside anisotropic wet etching and to prevent electrical interference from an ionic solution from entering the electrically-conductive piezoresistive layer. Lastly, a 30% potassium hydroxide (KOH) solution was used to release the microcantilever. Figure 1b depicts and SEM image of the complete piezoresistive microcantilever.

Figure 1c shows the overall package that consisted of a sensor chip, a poly-dimethylsiloxane(PDMS)-based microchannel, a channel bottom plate and a PCB with electronic components. A diced microcantilever chip was attached onto an electrical PCB and placed inside a microchannel. The microchannel was formed with a PDMS-based capping structure and a recessed silicon micromachined bottom plate. The microchannel with an embedded microcantilever sensor was designed in a rectangular cuboid 14.8 mm long, 2.4 mm wide and 100 µm high with a volume of 3.55 µL. The flow rate in the channel was 10 µL/min.

A single free-standing cantilever itself is very sensitive to temperature change. In this study, a change in temperature of 1 °C yields a microcantilever deflection or its associated signal that is far beyond the generated signal of the molecular interaction. As a consequence, a thermally-controlled system is needed for the measurement of the single free-standing microcantilever sensor. As shown in Figure 1d, the system was established to be heat insulated from the outside environment and to be temperature controlled within ±0.1 °C.

### 2.2. Surface Modification

To serve as a microcantilever biosensor, the physical sensing surface underwent several chemical and biochemical processes. First, a self-assembly monolayer (SAM) solution of 100 mM of 8-mercaptooctanoic acid (SH(CH_2_)_7_COOH) was injected into the microchannel of the microcantilever biosensor and was incubated for 12 h to achieve a dense alignment. The sulfur atom of one end in SAM covalently reacted with the gold surface, and the other functional terminal of the carboxylic group in SAM provided antibody attachment with a peptide bond. The solution was made in a mixture with 75% ethanol solvent. After, the linker molecules were covalently chemisorbed on the gold-coated surface of the microcantilever. This reactive self-assembled monolayer was activated using a mixed solution of 75 mg·mL^−^^1^ of EDC (*N*-ethyl-*N*'-dimethylaminopropyl carbodiimide) and 11.5 mg·mL^−^^1^ of NHS (*N*-hydroxysuccinimide) in an equal ratio. This solution was then injected into the microchannel for 40 min to activate the SAM biolinker. Three hundred microliters of the capture protein (monoclonal anti-valproic acid, mouse IgG1 kappa, Abcam, Cambridge, MA, USA) was then injected, followed by 100 μL of ethanolamine hydrochloride solution.

## 3. Results and Discussion

### 3.1. Detection of Valproic Acid

Valproic acid (C_8_H_16_O_2_) is an acidic chemical compound with a molecular weight of 144 Da. While the capture immunoglobin G (IgG) antibody has a molecular weight of around 150 kDa, this target drug of small molecules cannot be detected by the direct binding of the antigen (drug)-antibody interaction in label-free affinity optical SPR biosensors. Electrical detection based on nanomechanics depends solely upon the direct binding of drug-antibody recognition and biomolecular interaction, resulting in induced surface stresses and microcantilever deflection. To transform the physical cantilever into a biosensor, prior to drug detection, the cantilever underwent several major chemical and biochemical processes, including SAM coating and capture antibody immobilization. The processes may react with the cantilever to yield the respective response signals.

**Figure 2:**
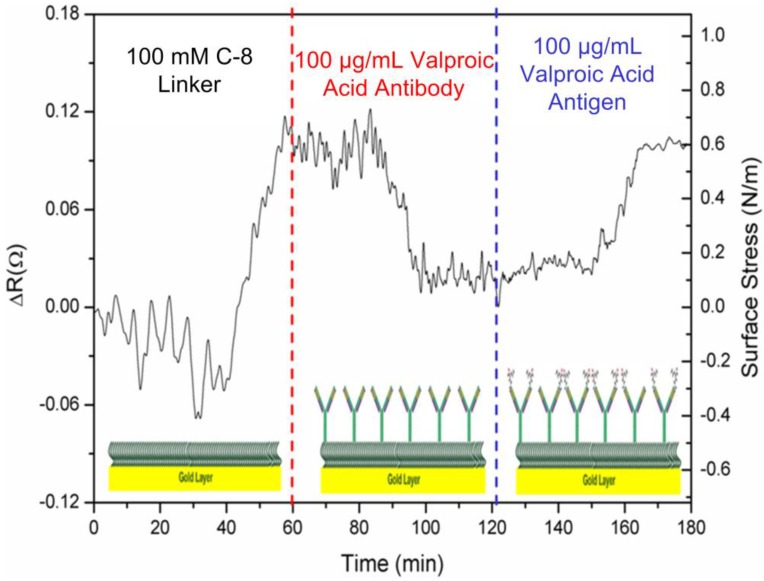
The detected signal pattern of a microcantilever with respect to the self-assembled monolayer coating, capture antibody immobilization and the antiepileptic drug,valproic acid.

In Figure 2a, the signal responses of the microcantilever can be obtained via deflections or be converted into induced surface stresses. First, the response signal of a cantilever was generated by the adsorption of 100 mM SAM biolinker, resulting in changes to resistance and surface stress of 0.12 Ω and 0.8 N·m^−^^1^ in compressive stress. During the adsorption of the alkanethiolate self-assembled monolayer (SAM) on the cantilever’s Au surface, the overall surface stresses originate from two types of interactions: surface charge redistribution and chain-chain interaction [19,20]. Meanwhile, surface charge redistribution gives rise to compressive surface stress, while chain-chain interactions produce tensile surface stress. Meanwhile, the stress induced by surface charge redistribution is about one order of magnitude greater than that of the chain-chain interactions [20]. As a result, the alkanethiolate SAM adsorption on the cantilever sensor’s gold surface generates a deflection of compressive surface stress.

In the second stage, a cantilever was deflected in the immobilization of 100 μg·mL^−^^1^ anti-valproic acid molecules. In commercial surface plasmon resonance biosensors, the capture antibody concentration for immobilization should be in the range between 10 μg·mL^−^^1^ and 200 μg·mL^−^^1^.In this study, a concentration of 100 μg·mL^−^^1^ was used.

The capture antibody molecules were bonded randomly onto the SAM layer. The induced change in surface stress was measured as 0.07 Ω and 0.55 N·m^−^^1^ in tensile stress, which displayed a bend-down deflection, indicating that capture antibody conformation and a molecule-level force rearrangement over a period of about 20 min achieved the required balance force on the microcantilever. After the functional SAM surface was deactivated or blocked, the immobilized antibody molecules on the microcantilever surface were then ready for interaction with the target drug. Therefore, the microcantilever was modified to be capable of sensing specific small molecules of the target drug.

In the last stage of target detection, the signal was generated by the specificity and interaction of the macromolecular capture antibodies with respect to 100 μg·mL^−1^valproic acid in PBS solution. It took approximately 20 min for the steady-state signal to achieve a molecular force equilibrium state. This direct binding gives rise to a considerable deflection of induced surface stresses. The continual deflection change prior to the equilibrium state could result from a gradual rearrangement of the antibody conformation. In this study, the antiepileptic valproic acid drug was first investigated using the microcantilever biosensor technique. In this measurement, the solution was controlled to create a friendly environment for drug-antibody direct binding. According to the isoelectric points (pI) of both target drug and capture antibody, the pH value of the solution maintained opposite charges for local attraction between the antibody and the drug target [21,22].

The isoelectric point is the pH value at which a particular molecule or surface carries no net electrical charge. Proteins are positively charged in a solution at a pH below the pI value and negatively charged above the pI value. In the proximity of the sensing surface, the appropriate pH solution helps both opposite charges attract the drug-antibody interaction. To select an appropriate pH environment for measurement, we investigated the pI values for both valproic acid and its antibody. According to a drug database, the isoelectric point is 4.8 [23].

A double sodium dodecyl sulfate polyacrylamide gel electrophoresis (dSDS-PAGE) was used to investigate the pI value of valproic acid antibodies. All antibodies shared a similar general structure, but the terminal tip of a Y-shaped antibody protein has a different binding site that allows the antibody to attach to its specific types of antigens. The Y-shaped protein comprises four peptide chains, including two identical heavy chains and two identical light chains connected by disulfide bonds. With different weights and charges that travel at different speeds, the dSDS-PAGE can separate molecules into a two-dimensional arrangement.In other words, at its pI value, the macromolecular capture antibody does not migrate in an electric field.

**Figure 3:**
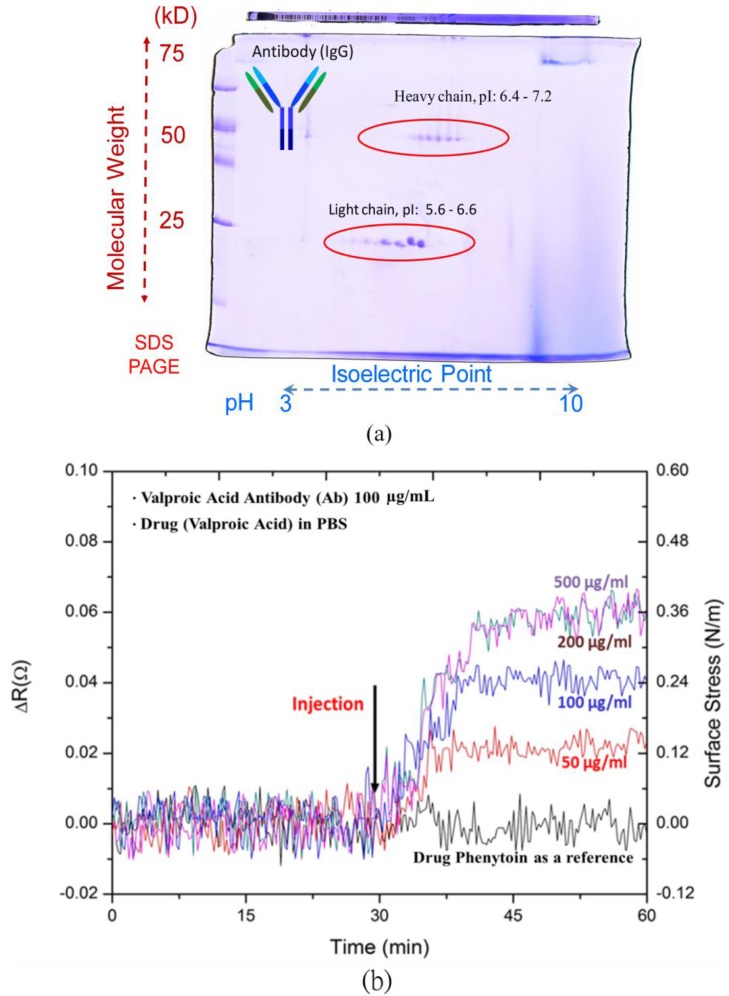
(**a**) A double sodium dodecyl sulfate polyacrylamide gel electrophoresis(dSDS-PAGE) for capture antibody; (**b**) detection of various valproic acid concentrations and the test of non-specific binding with another drug, phenytoin.

As shown in Figure 3a, the light chain denotes the molecular weight of 25 kDa distributed 5.6–6.6 in pI values, and the heavy chain with the molecular weight of 50 kDa exhibited 6.4–7.2 in its pI values. As a result of the dSDS-PAGE, the range of valproic acid antibody macromolecules was simply shown to be 5.6–7.2. To obtain opposite charges for the drug and antibody, the pH environment of the PBS solution was set at pH 5. Both the antibody and drug molecules exhibited opposite charges for attraction to enhance the binding capability of the microcantilever biosensors. Meanwhile, the antibody and the antiepileptic drug, valproic acid, respectively carried negative and positive charges in solution.

The results in Figure 3b show the resistance changes to the electrical responses of the microcantilever biosensors with respect to various concentrations of valproic acid between 50 μg·mL^−1^ and 500 μg·mL^−1^, covering a therapeutic range of 50–100 μg·mL^−1^.The resistance change of the steady-state response signals increased with the valproic acid concentration.

To verify specificity, we investigated small molecules of the commonly-used antiepileptic drug, phenytoin, which has a narrow reference range of 10–20 ug·mL^−1^ and also requires TDM. The response signal of the microcantilever biosensor was found to be negligible in the presence of 100 ug·mL^−1^ phenytoin in PBS solution. This ensured the high specificity of the microcantilever biosensors for drug detection in antiepileptic therapeutic drug monitoring.

### 3.2. Drug-Antibody Binding Capability and Measured Reproducibility

**Figure 4:**
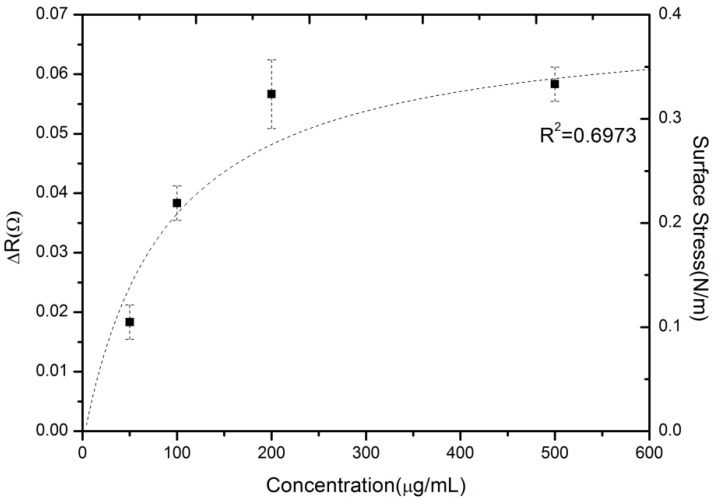
The reproducible, measured steady-state signals of various valproic acid concentrations.

A series of measurement experiments were conducted to examine the reproducibility of microcantilever detection and to characterize the binding property of drug-antibody interaction. Figure 4 shows a profile of the steady-state response signals as the valproic acid concentration increases. The average steady-state resistance changes with error bars at each concentration obtained for three tests. As the sensor device was treated as being for disposable use for a point-of-care platform or personalized diagnostics, the experiment was designed such that the sensor were not reusable. Hence, each sensor surface was not regenerated in the process, and thus, they were freshly constructed.

The dynamic range and sensitivity were also investigated by varying the valproic acid concentrations in PBS. The experimental results showed a distribution in a range between 50 and 500 μg·mL^−1^. This region was still within the clinically-relevant therapeutic range of valproic acid, which is important for pharmacokinetic or dynamic drug profiling and personalized medicine. A system noise value of around 0.005 Ω can be achieved in the resistance change using the average of all raw signals in a certain period. The estimated limit of detection (LOD), defined as the analyte concentration corresponding to a signal-to-noise ratio of three [24], was calculated at 45 μg/mL.Moreover, the sensor response sensitivity was obtained by (ΔR/R)/[concentration] as (1.28) × 10^−7^ (μg·mL^−1^)^−1^.

In addition, this plot may extract the quantitative biomolecular binding that accounts for drug-target binding events. This label-free microcantilever technique was used in previous studies [8,25] to obtain the binding affinity for small molecule drugs. The kinetics of biomolecular binding on microcantilever surfaces can be described using a simple Langmuir first-order scheme. The model assumes that the molecules are adsorbed at a fixed number of well-defined sites, each of which is energetically equivalent (a random process) and exhibits complete monolayer coverage when the surface reaches the saturation level. Meanwhile, the rate constant for binding kinetics (association) is given by k_a_, whereas the rate constant for unbinding kinetics (dissociation) is given by k_d_. The rate of binding between the capture antibodies and small molecules was obtained by random conjugation using the following equation [26].

(1) $$\frac{dN}{dt}=k_{a}\Phi (N)C_{s}-k_{d}N$$

where N is the number of binding molecules on the microcantilever surface as a function of time, C_s_ is the valproic acid drug concentration on the sensing surface and Φ(N) gives the number of remaining pairs available for binding. The total amount of analyte Φ(N)is expressed as (N_max_ − N) in terms of the maximum analyte binding capacity of the biochip surface. All concentration terms can then be expressed as the biosensor’s binding signal response, which is proportional to the surface protein concentration. It was assumed that the response (σ) of the microcantilever from the conjugation complex can effectively contribute to the generation of surface stress. In practice, the correlation can be described in an equilibrium state by an input of dN/dt = 0 and in terms of the saturation level in the equilibrium state to derive another form of kinetic basis system [26]:

(2) $$\sigma_{eq}=\frac{\sigma_{\max}C_{S}}{K_{D}+C_{S}}$$

Since σ_eq_ and σ_max_ respectively represent the equilibrium (steady state) and maximum values of surface stress, K_D_ is equal to k_d_/k_a_ and stands for the equilibrium dissociation constant or binding affinity. As σ_eq_ is set to be at σ_max_/2, K_D_ can be determined as being equal to C_s_. In this study, the binding affinity (K_D_) was calculated to be around 90 ± 21 µg·mL^−1^.In previous studies, the average binding affinities of valproic acid and its antibody were respectively measured at 9 µg·mL^−1^ (62.5 µmol·L^−1^) [27] and 66 µg·mL^−1^ (460 µmol·L^−1^) [28]. The present result lies in the same order of magnitude.

As shown in Figure 4, the maximum value of surface stress (σ_max_) can be assumed to be 0.4 N·m^−1^, and the binding affinity (K_D_) was set at 90 ± 21 µg·mL^−1^. Based on Equation (2), the equilibrium surface stress (σ_eq_) can be obtained as σ_eq_ = 0.4C_s_/(90 + C_s_). Figure 4 shows the equilibrium surface stress distribution of the dotted line as a function of concentration based on the Langmuir adsorption isotherm model. As a result, in Figure 4, the label-free microcantilever biosensor for small-molecule detection can be further studied to gain insight into the biomolecular binding event between small-molecule drug and macromolecular antibody.

### 3.3. Detection of Valproic Acid in Serum

The clinical effects of valproic acid are strongly correlation with serum drug concentration [29]. As the valproic acid molecules are highly (>90%) bound to serum proteins, the detection of free (unbound) drug concentrations in serum may be clinically useful and is less effective than that of total drug concentration. The serum carries proteins (mostly albumin, immunoglobulins, interferon,* etc.*), electrolytes, antibodies, antigens, hormones and drugs [30]. Moreover, a number of factors may alter serum protein concentrations, including liver disease, old age and pregnancy. To aid in the detection in fetal bovine serum (FBS), the surface coverage of the capture antibody could be increased on the microcantilever sensing area in such a complex serum environment. With the injection of the capture antibody concentration from 100 µg·mL^−1^ to 300 µg·mL^−1^ for high immobilization, the microcantilever sensing surface was increased to provide target drug molecules with more antibody molecules available for binding.

**Figure 5:**
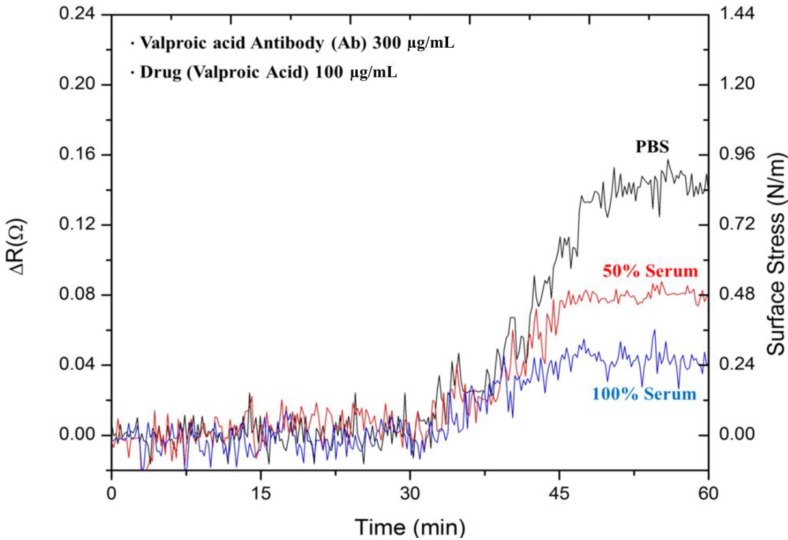
The measured results of 100 μg·mL^−1^ valproic acid in PBS, 50% serum and pure serum.

Figure 5 shows the signals responses for the detection of 100 µg·mL^−1^ valproic acid drug in three solutions of PBS, 50% FBS with 50% DI water and 100% FBS. As described earlier, these solutions were kept at pH 5 in solution. The response signals in the three environments exhibited similar profiles over the time period. In PBS, the microcantilever was significantly deflected. The response signals were considerably reduced in the presence of a complex serum environment. As expected, the resistance change of 0.04 Ω in 100% serum was even lower than that of 0.08 Ω in 50% serum. The surface stresses were 0.24 N·m^−1^ in 100% serum, a lower response than that of 0.48 N·m^−1^ in 50% serum. The presence of a complex environment, as well as relevant protein binding to the free valproic acid drug in serum considerably reduced the interaction between the antibody and the valproic acid.

### 3.4. Comparison Results

**Figure 6:**
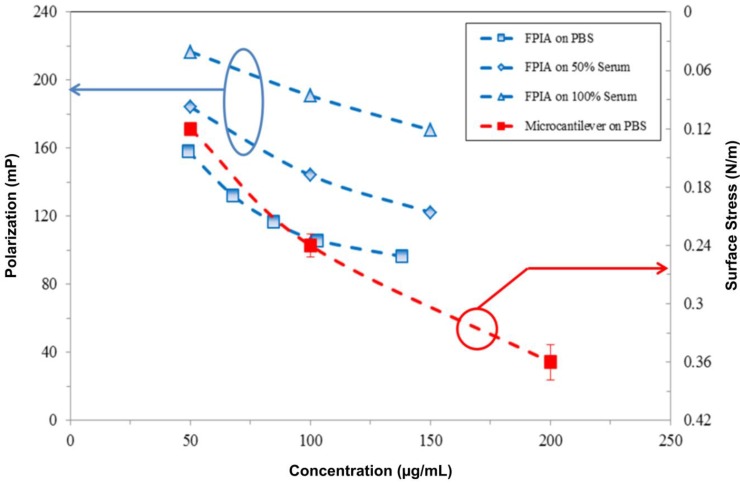
The similar tendency of the response signals obtained by the fluorescence polarization immunoassay (FPIA) and the present microcantilever biosensor in PBS, 50% serum and pure serum.

Figure 6 compares the results of valproic acid drug detection using the proposed piezoresistive microcantilever biosensor and clinical measurements using the fluorescence polarization immunoassay (FPIA). FPIAs are homogeneous, single-step assays suited for the high-throughput screening of large numbers of samples. In clinical environments, such assays require trained staff, milliliter-scale samples and reagent volumes and a turnaround time of almost one day to complete [15]. The deflection of a cantilever biosensor was induced by drug-antibody direct interaction and binding. FPIAs are based on the competition of fluorophore-labeled valproic acid with the free (unlabeled) valproic acid drug in a sample with respect to specific capture antibodies. Both labeled and unlabeled valproic acid molecules mixed with the antibody in the same solution revealed competition in terms of molecular binding. By increasing the concentration of unlabeled valproic acid molecules in a binding competition environment, we found that the signal exhibited a low polarization reading. Meanwhile, this fluorescence polarization is quantified as milli-polarization units, or mP.

In FPIA measurements, three samples of the antiepileptic drug, valproic acid, with concentrations of 50, 100 and 150 μg·mL^−^^1^ were respectively conducted in PBS, 50% and 100% fetal bovine serum. Prior to the measurement, the experiment of which the results measured by the FPIA exhibited a linear correlation with the given concentrations proved to be valid for the FPIA as a reference technique. Figure 6 shows the results of the FPIA based on the competition approach. Meanwhile, a low-polarization reading resulted in a high concentration of the target valproic acid drug. In a 100% serum environment, the plot was obtained in an approximate parallel shift by the FPIA, resulting in lower sensitivity than that in PBS. Moreover, the experiment with three samples in concentrations of 50, 100 and 200 μg·mL^−1^ in PBS was performed using the proposed single, free-standing piezoresistive microcantilever biosensor.

Figure 6 shows the similar trends between valproic acid concentrations measured in microcantilever and FPIA techniques. Though these two techniques employed different units, the signals decreased with increasing valproic acid concentrations. Likewise, a large amount of drug-antibody binding resulted in strong surface stresses over the microcantilevers. As a result, the valproic acid drug detection measured by the label-free, single, free-standing piezoresistive microcantilever biosensors showed a similar profile as that in the clinically-used FPIA.

## 4. Conclusions

Direct determination of small molecular valproic acid was first investigated by a single, free-standing, label-free piezoresistive microcantilever biosensor. The single, free-standing cantilever was manufactured by micromachining to be a flat and straight shape for detection. Measurements of the epileptic drug, valproic acid, were performed in PBS and in fetal bovine serum environment. The measured surface stresses were obtained in a concentration range of 50–500 μg·mL^−1^, which covered the clinically-relevant valproic acid therapeutic range of 50–100 μg·mL^−1^. The result of the proposed single, free-standing microcantilever biosensors showed a similar profile in valproic acid drug detection with that of the clinically-used FPIA. The reproducibility and valproic acid binding affinity results are valuable in the context of the development of antiepileptic medications using the label-free, miniaturized microcantilever biosensors.
